# Construction and validation of a nomogram for blood transfusion after open reduction and internal fixation (ORIF) of proximal humeral fractures in the elderly: a cross-sectional study

**DOI:** 10.1186/s12891-024-07661-1

**Published:** 2024-07-11

**Authors:** Lu-ying Chen, Ji-qi Wang, You-ming Zhao, Yong-zeng Feng

**Affiliations:** 1 Department of Medical Ultrasonics, The First Affiliated Hospital of Wenzhou Medical University, Shangcai Village Ouhai District, Wenzhou, 325000 Zhejiang China; 2 Department of Orthopaedics, The Second Affiliated Hospital and Yuying, Children’s Hospital of Wenzhou Medical University, 109# Xue Yuan Xi Road, Wenzhou, 325000 Zhejiang China

**Keywords:** Proximal humeral fractures, Blood transfusion, Risk factor, Open reduction and internal fixation, Nomogram, Prediction model

## Abstract

**Purpose:**

Few studies have focused on the risk factors leading to postoperative blood transfusion after open reduction and internal fixation (ORIF) of proximal humeral fractures (PHFs) in the elderly. Therefore, we designed this study to explore potential risk factors of blood transfusion after ORIF for PHFs. We have also established a nomogram model to integrate and quantify our research results and give feedback.

**Methods:**

In this study, we retrospectively analyzed the clinical data of elderly PHF patients undergoing ORIF from January 2020 to December 2021. We have established a multivariate regression model and nomograph. The prediction performance and consistency of the model were evaluated by the consistency coefficient and calibration curve, respectively.

**Results:**

162 patients met our inclusion criteria and were included in the final study. The following factors are related to the increased risk of transfusion after ORIF: time to surgery, fibrinogen levels, intraoperative blood loss, and surgical duration.

**Conclusions:**

Our patient-specific transfusion risk calculator uses a robust multivariable model to predict transfusion risk.The resulting nomogram can be used as a screening tool to identify patients with high transfusion risk and provide necessary interventions for these patients (such as preoperative red blood cell mobilization, intraoperative autologous blood transfusion, etc.).

## Introduction

Proximal humeral fracture (PHF) is a prevalent type of fracture in the elderly, accounting for about 5% of all fractures [1]. It is generally believed that patients with displaced and unstable PHFs need surgical treatment to obtain early postoperative functional recovery [2, 3]. There is a multitude of surgical treatments for PHFs; open reduction and internal fixation (ORIF) with stainless steel plate is undoubtedly the most commonly used [4, 5]. However, many related complications have been reported, among which postoperative blood transfusion is common and troublesome [6–8]. Cvetanovich et al [9]. have reported that the overall blood transfusion rate of patients with PHFs after ORIF was about 10.1%. A blood transfusion may lead to complications, such as surgical site infection, thrombosis, and cardiovascular events. At the same time, a blood transfusion may increase hospital stay and treatment costs [10–12]. It is worth mentioning that the elderly are more likely to have transfusion-related complications because of their low tolerance to blood transfusion. Therefore, it is necessary to strengthen the perioperative blood management.

Previous studies have described the correlation between blood transfusion and adverse events after PHF surgery, length of hospital stay, and delayed discharge [13]. Few studies have focused on the risk factors leading to postoperative blood transfusion. Therefore, we designed this study to explore potential risk factors of blood transfusion after ORIF for PHFs. We also established a nomogram model to integrate and quantify our research results and give feedback.

## Patients and methods

We obtained the approval of the Institutional Ethics Committee before the start of this retrospective study. The inclusion criteria were: (1) Age ≥ 65 years old; (2) The injury time is less than two weeks; (3) No history of previous injury to the shoulder joint; (4) No previous history of blood diseases (such as hemophilia and vitamin K deficiency); (5) No history of anticoagulant use; (6) Complete medical information. Exclusion criteria: (1) Open fracture; (2) Pathological fracture; (3) Loss to follow-up. From January 2020 to December 2021, 238 PHF patients received ORIF in our institution; 76 of them were excluded: 32 patients were younger than 65 years old, 15 patients lost to follow-up, 11 patients had a previous history of oral anticoagulant drugs, eight patients were combined with fractures in other parts, five patients had a history of rotator cuff injury on the same side, two patients had been injured for more than two weeks, two patients had pathological fractures, and one patient had open fractures. Finally, 162 patients met our inclusion criteria and were included in our study. All patients were operated on through the deltoid pectoralis approach, and a stable angle locking plate was selected as the internal fixator (Synthes Inc., Stratec Medical Ltd., Solothurn, Switzerland). All patients accepted the standard routine postoperative management plan. The X-ray examination was performed on the second day after the operation to evaluate the quality of fracture reduction. The follow-up plan is to recheck every other month within three months and every three months thereafter. According to our institutionalpolicy, all patients receive standardized rehabilitation and physical therapy on the first day after surgery and are shown functional exercise during each outpatient follow-up. According to our institution’s policy, each patient underwent blood testing on the first day after surgery, a blood transfusion is carried out when the patient’s hemoglobin is lower than 80 g/L or has ischemic symptoms (such as shock, chest pain, and shortness of breath).

## Patient characteristics

The assessed characteristics include the patient’s gender; age; body mass index (BMI); overweight status (BMI > 24 kg/m^2^); comorbid diseases (i.e., hypertension, diabetes); mechanism of injury: low-energy injuries (falls from standing height) and high-energy injuries (such as traffic accidents and falls from greater heights); fracture type (Neer’s classification [14] ); the presence of medial calcar support (the length of the posteromedial metaphyseal extension and the integrity of the medial hinge); [15, 16]. perioperative hematological indexes, such as preoperative hemoglobin (Hb), activated partial thromboplastin time (APTT), prothrombin time (PT), Thrombin time (TT), international normalized ratio (INR), D-Dimer, Platelet count, Fibrinogen levels, intraoperative blood loss (IBL), transfusion volume (TV) and postoperative hemoglobin; time to surgery; American Society of Anesthesiologists (ASA) scores; surgical duration; postoperative head shaft angle (HSA); postoperative length of stay (PLOS) and hospitalization expenses. Perioperative information was obtained from the institution’s electronic medical record system. ASA score, IBL, and surgical duration were obtained from the anesthesia records. The assessment of fracture type, medial calcar support, and HSA measurement was independently completed twice by two radiologists who did not participate in this study. If the assessment results of fracture type and medial calcar support differed, the conflict was resolved through discussion, and the mean value of the four results was taken for HSA.

## Statistical analysis

The statistical analyses were performed using SPSS for Windows software (ver. 19.0; SPSS Inc., Chicago, IL, USA). Means were compared using the independent samples t-test for normally distributed variables; otherwise, the Mann–Whitney U test was used for group comparisons, and qualitative variables were compared using the chi-square test. Univariate analysis assessed the association between different factors and blood transfusion. Then, multivariate logistic regression was performed to control for confounding effects. Predictor exclusion was continued until all predictors had p values less than 0.05, which was then defined as the final prediction model. Our study set the level of significance at 0.05 for all analyses.

## Results

Finally, our study included 162 elderly patients with PHFs, with 21 (12.9%) receiving blood transfusions after surgery. Table 1 describes the essential characteristics of the group of patients without blood transfusion and those with blood transfusion. We found that the proportion of overweight patients in the blood transfusion group was significantly higher, and they received surgical treatment earlier. The preoperative ASA score and the surgical duration were higher, and the PLOS was longer at a statistically significant level. Therefore, the total hospital cost of blood transfusion patients also increased significantly. Moreover, Table 2 shows the perioperative hematological indicators of the two groups of patients. We found that the preoperative fibrinogen levels of the patients in the blood transfusion group was significantly lower, and there was more IBL during the operation. Tables 3 and 4 summarize the univariate and multivariate analysis results of postoperative blood transfusion. The following factors were associated with an increased risk of blood transfusion after ORIF of PHFs: time to surgery, fibrinogen levels, IBL, and surgical duration.

**Table 1:** Differences in basic characteristics between the two groups

Characteristics	Non-transfused patients	Transfused patients	*p* value
Number of patients	141	21	
Gender			0.239
Male	25 (17.73%)	6 (28.57%)	
Female	116 (82.27%)	15 (71.43%)	
Age (Year)	71.65 ± 6.26	71.05 ± 4.66	0.675
BMI (Kg/m^2^)	22.45 ± 2.50	23.44 ± 3.30	0.106
Overweight (BMI > 24)			***0.030***
No	106 (75.18%)	11 (52.38%)	
Yes	35 (24.82%)	10 (47.62%)	
Hypertension			0.736
No	86 (60.99%)	12 (57.14%)	
Yes	55 (39.01%)	9 (42.86%)	
Diabetes			0.673
No	120 (85.11%)	17 (80.95%)	
Yes	21 (14.89%)	4 (19.05%)	
Mechanism of injury			0.553
Low-energy injuries	109 (77.30%)	15 (71.43%)	
High-energy injuries	32 (22.70%)	6 (28.57%)	
Fracture type			0.315
II	34 (24.11%)	2 (9.52%)	
III	59 (41.84%)	11 (52.38%)	
IV	48 (34.04%)	8 (38.10%)	
Medial calcar support			0.433
Yes	80 (56.74%)	10 (47.62%)	
No	61 (43.26%)	11 (52.38%)	
Time to surgery (d)	2.99 ± 1.09	1.81 ± 0.68	***< 0.001***
ASA score			***< 0.001***
1	77 (54.61%)	4 (19.05%)	
2	55 (39.01%)	6 (28.57%)	
3	9 (6.38%)	11 (52.38%)	
Surgical duration (min)	92.86 ± 7.82	107.90 ± 11.95	***< 0.001***
HSA	136.08 ± 4.89	136.71 ± 4.37	0.574
PLOS (d)	4.73 ± 1.78	6.13 ± 2.04	***0.028***
Costs (CNY)	33569.91 ± 3338.70	38144.33 ± 3075.05	***< 0.001***

BMI, body mass index; ASA score, American Society of Anesthesiologists score; HSA, head shaft angle; PLOS, postoperative length of stay

**Table 2:** Difference of perioperative hematological indexes between two groups

Characteristics	Non-transfused patients	Transfused patients	*p* value
Pre-Hb (g/L)	125.35 ± 10.76	127.14 ± 10.14	0.474
APTT (s)	34.08 ± 4.47	32.84 ± 3.16	0.221
PT (s)	13.05 ± 0.73	13.00 ± 0.38	0.742
TT (s)	16.22 ± 0.99	16.19 ± 1.14	0.887
INR	1.03 ± 0.07	1.03 ± 0.03	0.858
D-Dimer (ug/mL)	3.92 ± 6.16	2.83 ± 4.80	0.436
Platelet (*10^9^/L)	223.41 ± 70.82	208.33 ± 52.03	0.350
Fibrinogen (g/L)	4.23 ± 1.01	2.94 ± 0.41	***< 0.001***
IBL (ml)	203.90 ± 62.41	323.81 ± 76.84	***< 0.001***
TV (Units)	-	2.24 ± 1.30	-
Post-Hb (g/L)	92.36 ± 12.69	72.67 ± 9.71	***< 0.001***

Pre-Hb, preoperative hemoglobin; APTT, activated partial thromboplastin time; PT, prothrombin time; TT, Thrombin time; INR, international normalized ratio; IBL, intraoperative blood loss; TV, transfusion volume; Post-Hb, postoperative hemoglobin

**Table 3:** Univariate association for each risk factor and blood transfusion

Characteristics	Correlation coefficient	*p* value
Gender	0.093	0.241
Overweight	0.171	***0.030***
Mechanism of injury	0.047	0.556
Hypertension	0.026	0.738
Diabetes	0.064	0.808
Fracture type	0.084	0.287
Medial calcar support	-0.062	0.436
ASA	0.395	***< 0.001***
Age	0.007	0.929
BMI	0.142	0.071
Time to surgery	-0.364	***< 0.001***
Pre-Hb	0.122	0.121
APTT	-0.097	0.219
PT	-0.026	0.739
TT	-0.012	0.878
D-Dimer	0.022	0.785
Platelet	0.003	0.972
Fibrinogen	-0.449	***< 0.001***
IBL (ml)	0.502	***< 0.001***
Surgical duration	0.384	***< 0.001***
HSA	0.030	0.705

ASA, American Society of Anesthesiologists score; BMI, body mass index; Pre-Hb, preoperative homoglobin; APTT, activated partial thromboplastin time; PT, prothrombin time; TT, Thrombin time; INR, international normalized ratio; IBL, intraoperative blood loss; HSA, head shaft angle

**Table 4:** Risk factors for blood transfusion (binary logistic regression models)

Characteristics	OR (95% CI)	*p* value
Overweight	2.06 (0.21, 20.02)	0.534
ASA	3.10 (0.49, 19.81)	0.231
Time to surgery	0.15 (0.03, 0.95)	***0.041***
Fibrinogen	0.01 (0.01, 0.30)	***0.008***
IBL	1.02 (1.00, 1.04	***0.032***
Surgical duration	1.13 (1.06, 1.42)	***0.006***

ASA, American Society of Anesthesiologists score; IBL, intraoperative blood loss

**Fig. 1:**
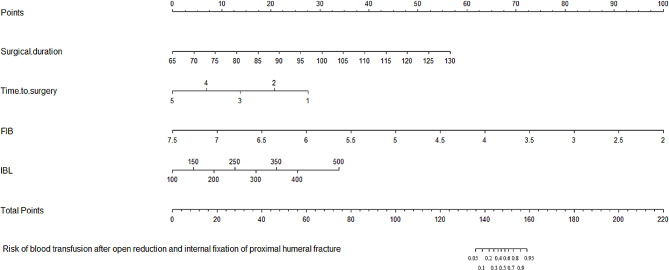
A nomograph for predicting the incidence of transfusion in elderly proximal humeral fractures after open reduction and internal fixation. The absolute value of each risk factor is correlated vertically to the corresponding value of the points scale in the first line of the nomograph, the individual adjusted values are then added together and the sum is expressed as a percentage of the total points scale at the bottom line of the nomograph, and this value represents the blood transfusion risk. For example, a 70-year-old female patient with a PHF underwent surgery the day after the injury. Her fibrinogen levels index before surgery was 3.0 g/L, the surgical duration was 100 min, and the IBL was about 350 ml. In our model, her time-to-surgery score is 20, her fibrinogen levels score is 82, her surgical duration score is 30, her IBL score is 22, her total score is 154, and the corresponding blood transfusion risk is 88%

**Fig. 2:**
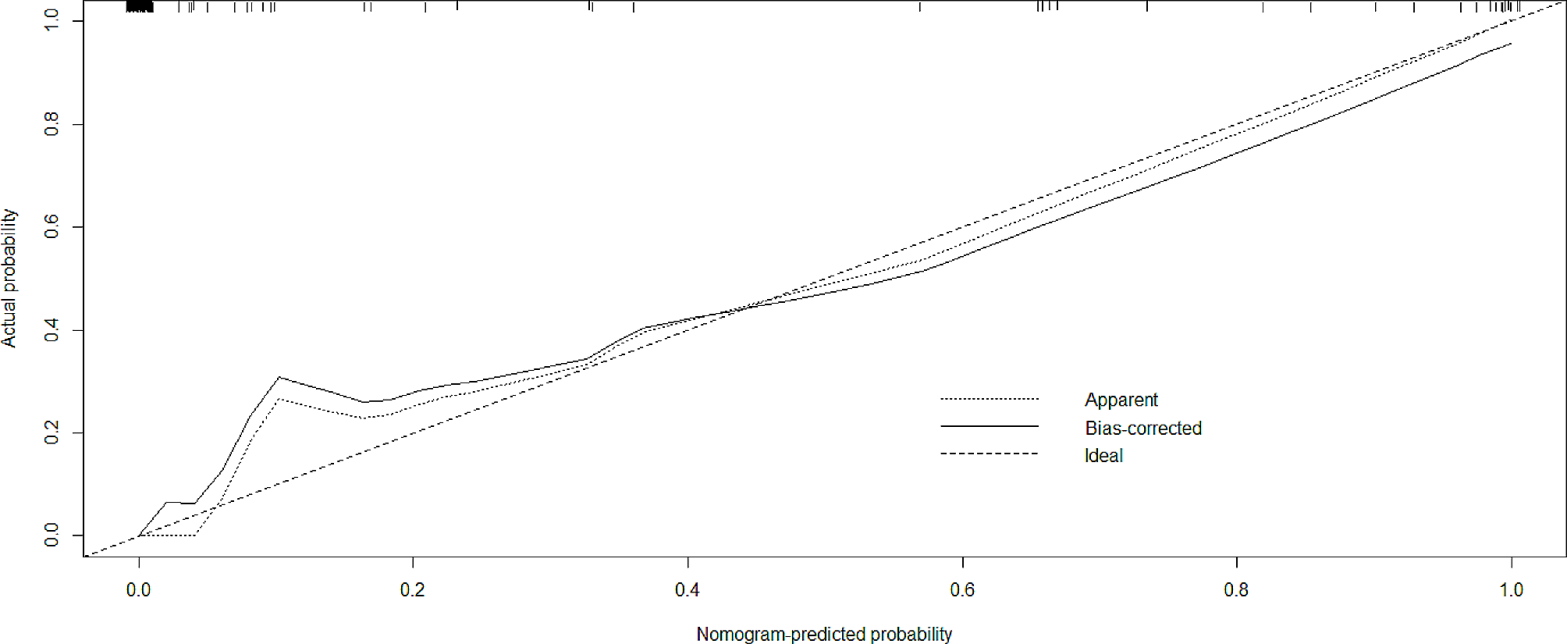
Calibration curve of nomograph prediction of dislocation in elderly proximal humeral fractures after open reduction and internal fixation

We then established a nomograph (Fig. 1) to predict the risk of blood transfusion after surgery, which quantifies each risk factor as a score, and the final risk probability is calculated as the percentage of the sum of the individual scores compared to the total maximum score of 220. For example, a 70-year-old female patient with a PHF underwent surgery the day after the injury. Her fibrinogen levels index before surgery was 3.0 g/L, the surgical duration was 100 min, and the IBL was about 350 ml. In our model, her time-to-surgery score is 20, her fibrinogen levels score is 82, her surgical duration score is 30, her IBL score is 22, her total score is 154, and the corresponding blood transfusion risk is 88%. The C-index (Concordance index, an indicator used to evaluate the predictive ability of a model, with a value close to “1”, indicating that the model is more accurate) of the model is 0.991, consistent with “excellent” calibration. Figure 2 shows the calibration curve. The average absolute error between the predicted and actual values is 0.017, and the expected risk is close to the substantial risk.

## Discussion

Proximal humeral fractures in the elderly are a significant public health problem. There are many complications after ORIF, such as superficial infection, blood transfusion, reduction loss, varus malunion, and even reoperation [13, 17, 18]. Among the most common and troublesome is blood transfusion, which is especially important because anemia is associated with increased mortality [19]. Previous studies have showed that blood transfusions are costly and might be associated with complications, such as surgical site infection and thrombotic and cardiac events; moreover, it may increase the length of hospital stay [10, 12]. Our results argee with previous studies’ conclusions, which suggested that elderly patients who received a blood transfusion have increased incidence of adverse events and postoperative length of stay and hospitalization costs. Thus, surgeons should perform more rigorous postoperative hemoglobin monitoring after surgery, and it is essential to clarify the impact of risk factors for blood transfusion and early intervention.

We were surprised to find that patients with shorter time to surgery were related to higher blood transfusion rates, which coincided with the results of the study of Malcherczyk et al [20]. In addition, patients with low preoperative fibrinogen levels were associated with a higher risk of blood transfusion, but preoperative platelet values did not reflect this relationship. Fibrinogen levels is a blood coagulation factor that can counteract hyperfibrinolysis and is closely related to bleeding. The lower the fibrinogen levels is, the more serious the bleeding tendency is [21]. Moreover, we believe that surgical trauma can cause stress inflammatory reactions in the different body tissues, leading to hyperfibrinolysis, which may lead to more bleeding. This phenomenon may be pronounced in patients with early surgery after injury [22]. The surgical duration and IBL were independent risk factors of blood transfusion after ORIF in our study, and we considered that it might be related to the tourniquet not being used during the surgery of PHF. Longer surgical duration usually represents more IBL, and our preliminary data suggested a statistically higher amount of IBL in the transfusion group. Moreover, the increased surgical duration led wound sites to more prolonged exposure to the operating room surroundings, which might result in higher superficial infection rates, even though our research does not reflect this [23]

We know that many transfusion-related complications in elderly patients with proximal humeral fractures after ORIF have been reported. Still, our study is the first prediction model focusing on calculating the blood transfusion risk in patients with proximal humeral fracture, and we use nomographs to express these risk factors intuitively. The prediction model can respond to the perioperative characteristics of patients, so it can be used as a screening tool to identify and personalize recommendations and treatment. It is worth mentioning that blood transfusion would prolong the length of hospital stay and aggravate the financial burden; this problem should not be ignored when treating elderly PHF with ORIF. In addition, for those elderly patients with potential risk factors, especially at the same time combined with multiple injuries or other severe medical comorbidities, it is necessary to strengthen blood management during the perioperative period, such as controlled hypotension, intravenous iron, intraoperative blood salvage, and administration of antifibrinolytic agents.

Our study carried several limitations. First, we have performed a retrospective analysis, and the samples in our study were relatively small, which can not exclude the effect of other potential factors on the results (i.e., osteoporosis and bone mineral density). Furthermore, early functional outcomes and long-term follow-up data were not included; including such data would have improved our study. Finally, our hospital’s database does not include details about preoperative blood management, which may cause bias in the results. So, prospective studies with larger samples are required to validate our findings further.

## Conclusions

The incidence of blood transfusion after ORIF in elderly patients with a proximal humeral fracture is related to the following factors: time to surgery, fibrinogen levels, IBL, and surgical duration. The resulting nomogram can be used as a screening tool to identify patients with high transfusion risk and provide necessary interventions for these patients (such as preoperative red blood cell mobilization, intraoperative autologous blood transfusion, etc.).

## Data Availability

The datasets analyzed in the study are available from the corresponding author upon reasonable request.
